# Serum CXCL9 as a potential marker of Type 1 inflammation in the context of eosinophilic asthma

**DOI:** 10.1111/all.13924

**Published:** 2019-06-17

**Authors:** Takehiro Hasegawa, Takahiro Okazawa, Hitoshi Uga, Hirokazu Kurata, Akio Mori

**Affiliations:** ^1^ Central Research Laboratories Sysmex Corporation Kobe Japan; ^2^ National Hospital Organization Sagamihara National Hospital, Clinical Research Center Sagamihara Japan

**Keywords:** bronchial asthma, CXCL9, eosinophilic inflammation

**Abbreviations**
AHR: airway hyper‐responsiveness
BALF: Bronchoalveolar lavage fluid
CS: corticosteroids
DHBC: diseased human bronchial epithelial cells
EA: eosinophilic asthma
ELISA: enzyme‐linked immunosorbent assay
FEV: forced expiratory volume
HC: healthy control
NEA: noneosinophilic asthma
NHBC: normal human bronchial epithelial cells

To the Editor,

CXCL9 (MIG), CXCL10 (IP‐10), and CXCL11 (i‐TAC) are considered Th1‐type‐chemokines (T1 chemokines), as they are produced by airway epithelial cells with the stimulation of IFN‐γ.^1^ However, in vitro studies indicated that T1‐chemokines are involved in bronchial asthma which is generally considered a T2 disease, as they strongly enhance adhesion and release of granule proteins by eosinophils.^2^ Moreover, in vivo study using CXCR3‐knockout (KO) mice indicated that T1‐chemokines pathway is significantly involved in eosinophilic inflammation and airway hyper‐responsiveness (AHR).^3^ We investigated potential usefulness of serum CXCL9 as a biomarker of eosinophilic asthma.

We collected serum samples from 74 doctor‐diagnosed asthma patients and 19 healthy controls (HC) (Table S1), and measured CXCL9 and IL‐25, as previously described,^4^ using the DuoSet ELISA Development Systems (R&D Systems). Patients with asthma displayed either AHR evaluated by serial inhalation of acetylcholine and histamine or bronchial reversibility evaluated by inhalation of salbutamol. Patients with asthma were classified into eosinophilic (EA) or noneosinophilic asthmatic (NEA) groups, based on the presence or absence of eosinophils in the sputum stained by Hansel's method. Next, patients with asthma were classified into AHR‐positive and AHR‐negative patients, based on the inhalation challenge of serial dilution of acetylcholine or histamine. Moreover, patients with asthma were classified into atopic and nonatopic asthma groups, based on the existence of IgE antibodies against at least one perennial allergens. The study was approved by the research and ethics committees of Sagamihara National Hospital and Sysmex Corporation. All the study procedures were performed in accordance with the Declaration of Helsinki. Written informed consent was obtained from all patients.

Serum CXCL9 concentrations were significantly higher in patients with asthma than in healthy controls (Figure 1A), as well as in EA than in NEA (Figure 1B). On the other hand, no significant difference was observed between HC and NEA. CXCL9 concentrations of AHR‐positive patients were significantly higher than those of AHR‐negative patients (Figure 1C). In EA, acute exacerbation was significantly more common in patients whose CXCL9 concentrations were higher than the HC distribution (Figure 1D). Moreover, CXCL9 concentrations were negatively correlated with %FEV_1_ values (Figure 1E). These results collectively suggest that CXCL9 is involved in the pathogenesis of asthma.

**Figure 1:**
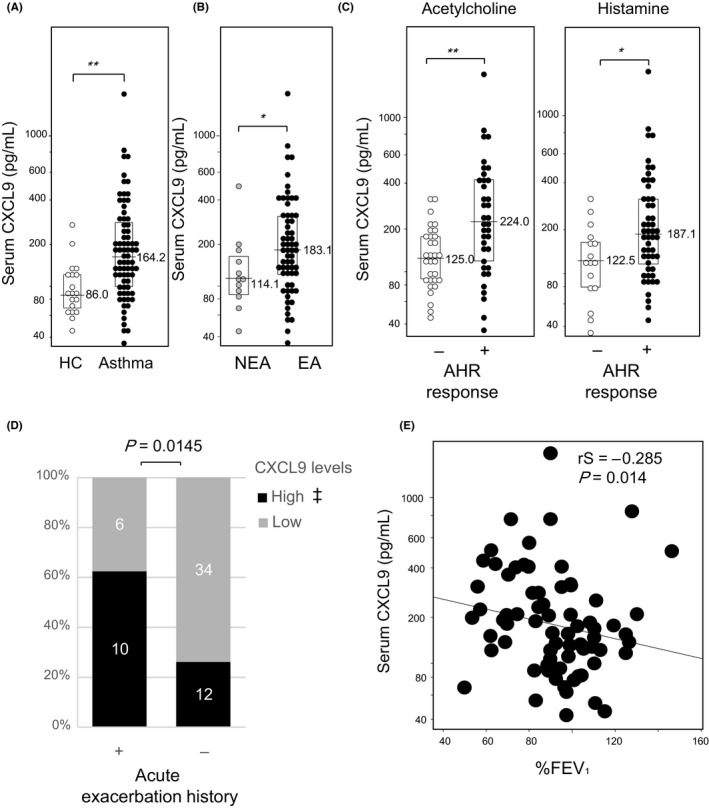
Serum CXCL9 concentrations reflect clinical characteristics of patients with asthma. A, Healthy controls (HC, open) and asthma (closed); B, eosinophilic asthma (EA, closed) and noneosinophilic asthma (NEA, gray); C, patients who showed more than (closed) or less than (open) 20% decrease in FEV_1_ up to 20 mg/mL acetylcholine (left) or up to 10 mg/mL histamine (right) challenge tests were defined as AHR‐positive or AH‐negative, respectively. Results are presented as individual data points with medians (bars) and interquartile ranges (boxes). Median values are indicated next to the bars. *P*‐values were calculated by the Mann‐Whitney *U* test. *: *P* < 0.05, **: *P* < 0.005; D, Relation between CXCL9 levels and acute exacerbation history; *P*‐value was calculated by Fisher's exact test. Exacerbation was defined based on systemic steroid usage within one year after blood collection. ‡: Cutoff value was defined by 2SD of HC distribution (229 pg/mL). E, Correlation was analyzed between %FEV_1_ and CXCL9 concentrations by Spearman's rank correlation

To further characterize EA and NEA groups, T2 markers such as pro‐Th2 cytokine IL‐25, blood eosinophil ratio, and serum total IgE were compared. These markers of EA were significantly higher than those of NEA, indicating that our EA population represented T2 phenotype (Figure S1). CXCL9 concentrations were significantly correlated with CXCL10 (Figure 2A), but not correlated at all with T2 markers such as IL‐25, blood eosinophil ratio, and serum total IgE (Figure 2B‐D). In addition, CXCL9 concentrations were not significantly different between atopic and nonatopic asthma (Figure S2D).

**Figure 2:**
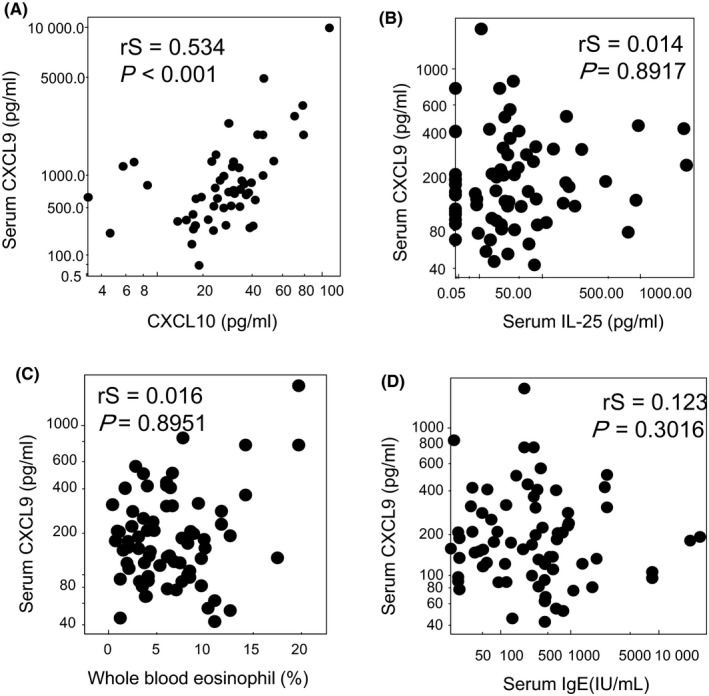
Relationship between serum CXCL9 concentrations and T1/T2 markers in patients with asthma; correlations between CXCL9 concentrations and CXCL10, a T1 marker (A), IL‐25, a pro‐Th2 cytokine (B), whole blood eosinophil ratio (C), and total serum IgE (D) were analyzed by Spearman's rank correlation

Both EA and atopic asthma belong to T2 asthma phenotypes, but might result from distinct pathophysiological processes.^5^ T2 markers including IL‐25 concentrations, blood eosinophil ratio, and serum total IgE were significantly elevated in both EA and atopic asthma (Figures S1 and S2), suggesting that both EA and atopic asthma groups in our current study represented T2 inflammation.^4^ However, our investigation clearly demonstrated that CXCL9, a T1‐chemokines, was significantly elevated in EA (Figure 1). In addition, CXCL9 concentrations were related to AHR, acute exacerbation, and lung function (%FEV1). On the other hand, it was not correlated with T2 markers like eosinophils, total serum IgE, and IL‐25 (Figure 2B‐D). These results collectively indicate that T1 inflammation might also contribute to the pathophysiology of EA in addition to T2 inflammation.

CXCL9 concentrations were not significantly increased in atopic asthma, in which T2 markers were clearly elevated (Figure S2 and our published article^4^). CXCL9 concentrations were significantly correlated with other T1‐chemokines such as CXCL10 (Figure 2A) and CXCL11 (data not shown), indicating that CXCL9 concentrations reflect T1 inflammation. CXCL9 concentrations were significantly elevated in nonatopic EA (Figure S2E), suggesting that T1 inflammation might contribute to eosinophilic inflammation of nonatopic asthma condition. In line with this notion, Modena et al performed airway epithelial cell gene expression analysis in 155 severe asthma research program subjects and identified T1 inflammation associated with increased T2 gene expression in a subgroup of patients with severe asthma.^6^ It was reported that the concentration of T1‐chemokines and the number of neutrophil and eosinophils count in the sputum samples were well correlated.^7^ The lack of correlation between the serum CXCL9 concentration and the number of blood neutrophils may be due to the different distribution of the samples or dilution of chemokines in the blood (Figure S3).

IFN‐γ induced airway epithelial cells to produce CXCL9 which was further enhanced by co‐culture with eosinophils.^1^ CXCL9 production by airway epithelial cells was induced by IFN‐γ and synergistically enhanced by eosinophilic cell line and TNF‐α (Figure S4). Min Xie et al reported IL‐27 and IL‐13 induced CXCL9 production by airway epithelial cells.^8^

Animal AHR models showed that CXCL9 was expressed at the site of airway inflammation.^9^ BALF eosinophilia was attenuated in CXCR3‐KO mice,^3^ clearly indicating the crucial role of CXCL9‐CXCR3 axis in airway eosinophilic inflammation. In humans, sputum concentrations of CXCL9 and CXCL10 were elevated in patients with high numbers of eosinophils and neutrophils in their sputum.^7^ It was also reported that the airway smooth muscle and submucosa of patients with asthma express CXCL10 with CXCR3‐expressing mast cell accumulation.^10^

In patients with sarcoidosis, CXCL9 and CXCL10 concentrations in the serum were well correlated with those in BALF.^11^ Serum and BALF CXCL10 concentrations were higher in the acute hypersensitive pneumonitis and cellular nonspecific interstitial pneumonia patients, in which CXCR3‐expressing T1 cells migrated into the lung parenchyma, suggesting serum concentrations of T1‐chemokines reflect inflammation of respiratory organs.^12^ It is recently reported that CXCL10 expression in the airway reflects resistance to corticosteroids (CS) treatment. CS enhanced T1 inflammation and made the disease worse in those patients.^13^ The distribution of CXCL9 concentrations in our patients seems somewhat broad (Figure 1), which is consistent with the reports previously made on IL‐27 and CXCL10.^7^, ^13^ Several possibilities including that CXCL9 low EA patients might consist of IL‐25 high patients seem intriguing and warrant further investigation.

Our findings collectively indicated that T1 as well as T2 inflammation is involved in eosinophilic asthma, and serum CXCL9 may be a good biomarker of T1 inflammation in EA. Serum CXCL9 might provide a good means to properly detect T1 inflammation in asthma patients without performing invasive biopsy or bronchoscopy and may be suitable for classification of T1 inflammatory endotypes.

## CONFLICTS OF INTEREST

T. Hasegawa, T. Okazawa, H. Uga and H. Kurata are employed by Sysmex Corporation. A. Mori declares no conflicts of interest associated with this manuscript.

## FUNDING INFORMATION

This work was funded by Sysmex Corporation.
