# Supplementary figures and images for: Serum CXCL9 as a potential marker of Type 1 inflammation in the context of eosinophilic asthma
Source: Allergy. 2019 Jun 17;74(12):2515–8. doi: 10.1111/all.13924 (PMC6972734; doi:10.1111/all.13924)

Hasegawa et al. Serum CXCL9 is a T1 inflammation marker in asthma

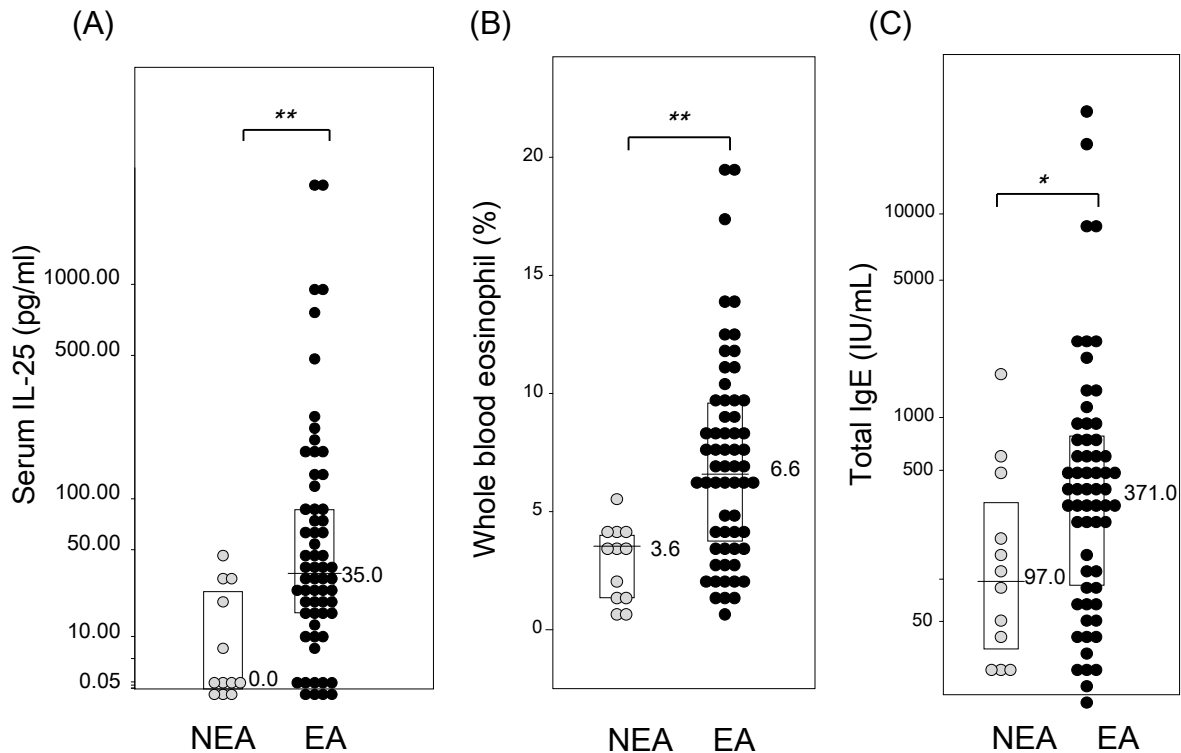

FIG.S-1

Supplement: Supplementary file 1 [file ALL-74-2515-s001.pdf]

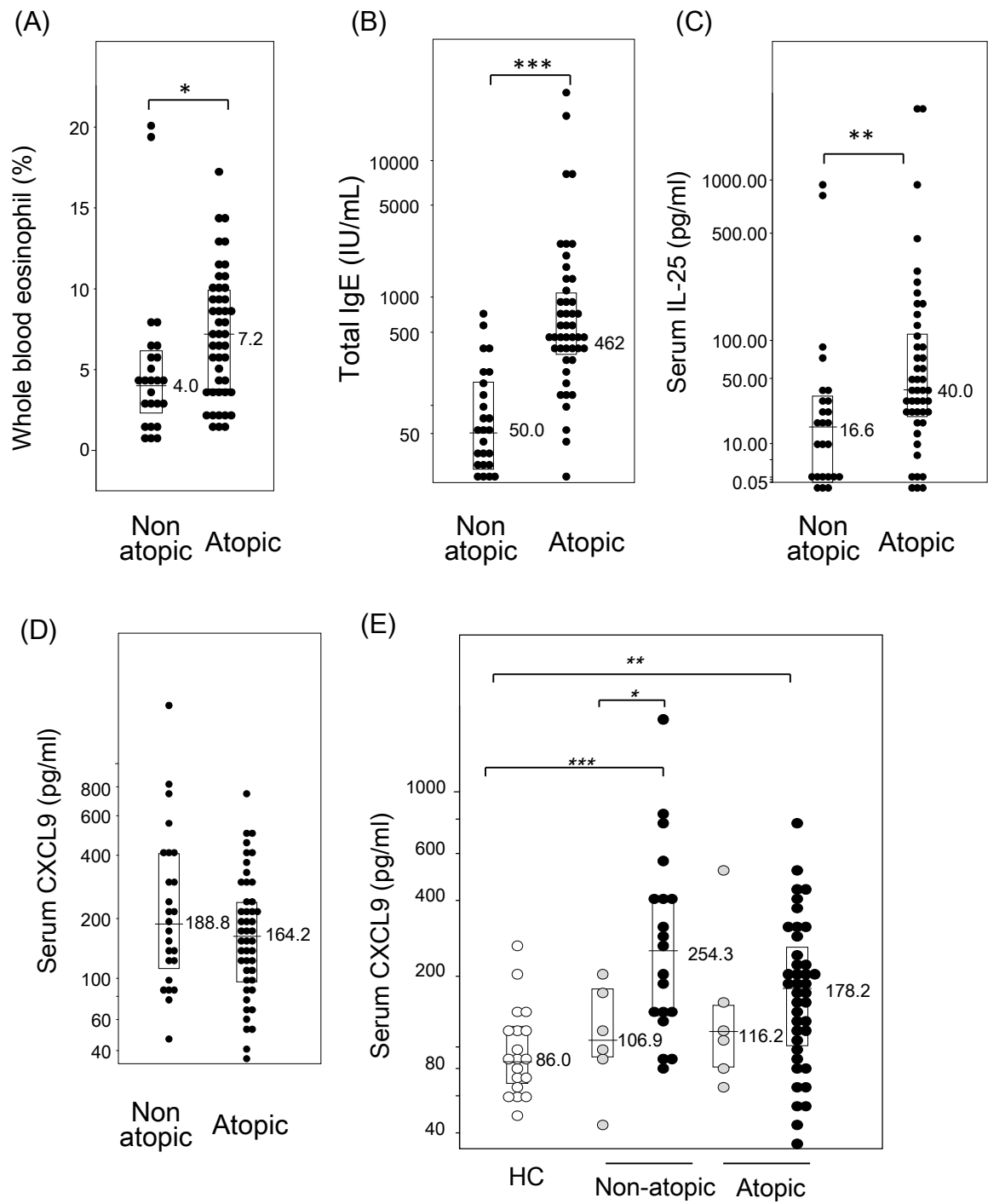

FIG.S-2

Supplement: Supplementary file 2 [file ALL-74-2515-s002.pdf]

Hasegawa et al. Serum CXCL9 is a T1 inflammation marker in asthma

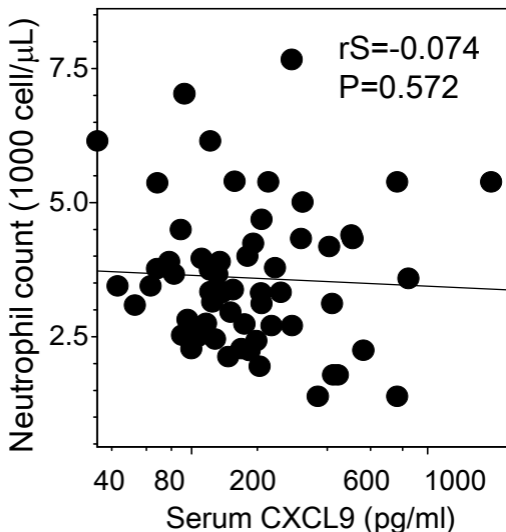

FIG.S-3

Supplement: Supplementary file 3 [file ALL-74-2515-s003.pdf]

Hasegawa et al. Serum CXCL9 is a T1 inflammation marker in asthma

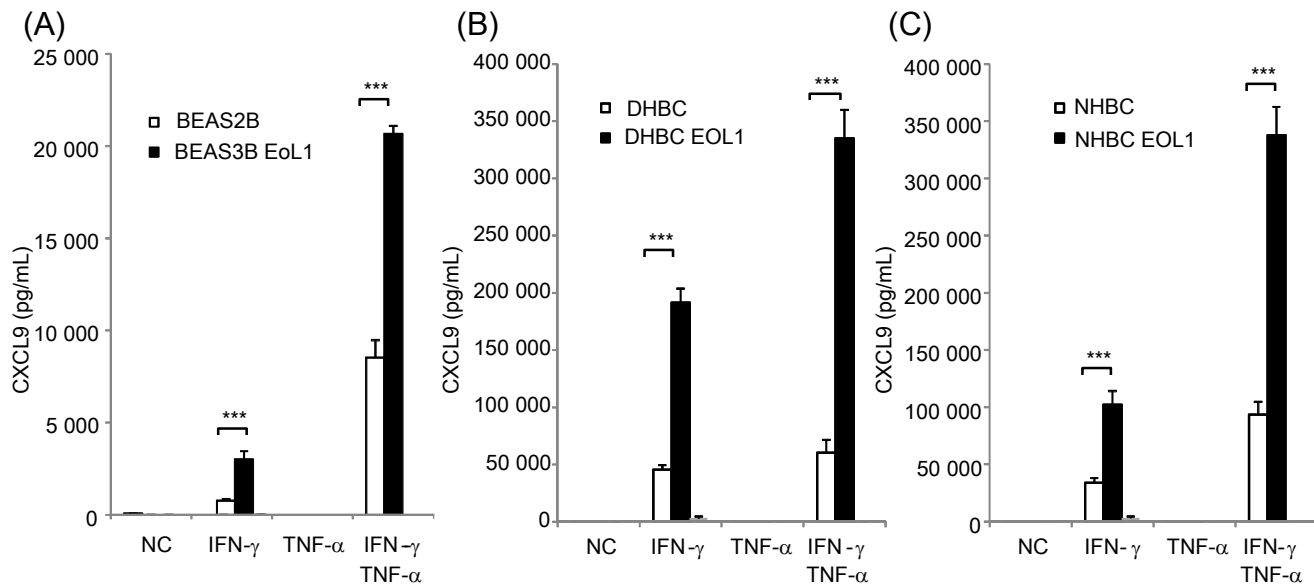

FIG.S- 4

Supplement: Supplementary file 4 [file ALL-74-2515-s004.pdf]
