# Supplementary material for: Serum CXCL9 as a potential marker of Type 1 inflammation in the context of eosinophilic asthma
Source: Allergy. 2019 Jun 17;74(12):2515–8. doi: 10.1111/all.13924 (PMC6972734; doi:10.1111/all.13924)
Supplement: Supplementary file 5 [file ALL-74-2515-s005.docx]

**Online supplement**

Letter to the Editor

Serum CXCL9 as a potential marker of Type 1 inflammation in the context of eosinophilic asthma

Takehiro Hasegawa PhD,^1^ Takahiro Okazawa, PhD,^1^ Mina Yabusaki, MSc,^1^ Hitoshi Uga, PhD,^1^ Hirokazu Kurata, MD, PhD,^1^ Akio Mori, MD, PhD^2^

1. Central Research Laboratories, Sysmex Corporation, Kobe, Japan

2. National Hospital Organization, Sagamihara National Hospital, Clinical Research Center, Sagamihara, Japan

Address correspondence and reprint requests to: Dr. Takehiro Hasegawa

Central Research Laboratories, Sysmex Corporation, 4-4-4, Takatsukadai, Nishi-Ku, Kobe, 651-2271, Japan

Email: Hasegawa.Takehiro@sysmex.co.jp

Tel: +81 78 992 5988

Fax: +81 78 992 3284

| **TABLE S-1** Demographic and clinical data | |  |  |  |  |  |  |  |  |  | |  |  |  |  |  | |
| --- | --- | --- | --- | --- | --- | --- | --- | --- | --- | --- | --- | --- | --- | --- | --- | --- | --- |
|  | **NEA^a^** | | **EA^b^** | | | | | | | | **HC^c^** | | | | | |  |
| Number (female/male) | 12 (6/6) | | 62 (34/28) | | | | | | | | 19 (9/10) | | | | | |  |
| Age (mean ±SD) | 57.7±16.9 † | | 55.8±15.7 † | | | | | | | | 42.1±9.8 † | | | | | |  |
| Atopic asthma^d^ (N) | 6 | | 40 | | | | | | | | N/A | | | | | |  |
| FEV_1_% predicted (mean ±SD) | 102.8±21.5 | | 89.7±19.3 | | | | | | | | N/D | | | | | |  |
| AHR^e^  (positive/negative) |  | |  | | | | | | | |  | | | | | |  |
| Acetylcholine | 3/8 | | 34/23 | | | | | | | | N/D | | | | | |  |
| Histamine | 7/4 | | 45/12 | | | | | | | | N/D | | | | | |  |
| Exacerbation^f^ (N) | 1 | | 11 | | | | | | | | N/D | | | | | |  |
| Severity^g^ (N) |  | |  | | | | | | | |  | | | | | |  |
| Mild intermittent | 6 | | 19 | | | | | | | | N/A | | | | | |  |
| Mild persistent | 1 | | 23 | | | | | | | | N/A | | | | | |  |
| Moderate persistent | 5 | | 17 | | | | | | | | N/A | | | | | |  |
| Severe persistent | 0 | | 2 | | | | | | | | N/A | | | | | |  |
| Inhaled corticosteroids (N) | 12 | | 60 | | | | | | | | N/A | | | | | |  |
| Oral corticosteroids (N) | 0 | | 1 | | | | | | | | N/A | | | | | |  |

^a^Non-eosinophilic asthma; ^b^Eosinophilic asthma; ^c^Healthy control; ^d^Atopic asthma patients have serum IgE antibody against at least one perennial airborne allergen;  ^e^Patients who showed more than 20% decrease FEV_1_ by serial inhalation of up to 20 mg/mL acetylcholine or 10 mg/mL histamine were defined as “Positive”. ^f^Exacerbation was defined by the deterioration accompanied with the use of systemic corticosteroids. ^g^Severity was defined according to GINA guideline. * p<0.05 from Mann-Whitney U-test; † p<0.05 from Kruskal–Wallis test;　N/A: not applicable; N/D: not determined.

**FIGURE LEGENDS**

**FIGURE S-1** Serum IL-25, whole blood eosinophil ratio, and serum total IgE of EA and NEA; Serum IL-25 concentration(A), whole blood eosinophil ratio (B), and median serum total IgE (C) were compared between NEA (gray) and EA (closed). Results are presented as individual data points with medians (bars) and interquartile ranges (boxes). Median values are indicated next to the bars.　P-values were calculated by Mann-Whitney U-test. *: P<0.05, **: P<0.005, ***: P<0.0005

**FIGURE S-2** Comparison of T2 markers and CXCL9 concentrations of atopic and non-atopic asthma; Whole blood eosinophil ratio (A), serum total IgE (B), pro-Th2 cytokine IL-25 (C), and CXCL9 concentrations (D) were compared between atopic and non-atopic asthma. (E) CXCL9 concentrations of NEA (gray) and EA (closed) in atopic and non-atopic asthma are shown. Results are presented as individual data points with medians (bars) and interquartile ranges (boxes). Median values are indicated next to the bars. P-values were calculated by the Mann-Whitney U-test. *: P<0.05, **: P<0.005

**FIGURE S-3** Relationship between serum CXCL9 concentrations and whole blood neutrophil count; Correlation between CXCL9 concentrations and whole blood neutrophil count was analyzed by Spearman’s rank correlation.

**FIGURE S-4** Induction of CXCL9 release by human airway epithelial cells upon interaction with human eosinophilic cell lines (EoL1) (A) BEAS-2B, (B) Diseased Human Bronchial Epithelial Cells (DHBC-As), and (C) Normal Human Bronchial Epithelial Cells (NHBC) (4 x 10^4^ cells/well) were cultured with or without a human eosinophilic cell line (EoL1) (4 x 10^4^ cells/well) for 36 hrs. in the presence of IFN-γ (100 ng/ml) or TNF-α (100 ng/ml) in a 24-well plate. CXCL9 concentrations in culture supernatants were assayed by a specific ELISA. Results are expressed as the mean (pg/ml) ± 2SD from three independent experiments. All p-values were calculated by Mann–Whitney U test. ***: P<0.0005
